# Retinoic Acid Ameliorates Pancreatic Fibrosis and Inhibits the Activation of Pancreatic Stellate Cells in Mice with Experimental Chronic Pancreatitis via Suppressing the Wnt/β-Catenin Signaling Pathway

**DOI:** 10.1371/journal.pone.0141462

**Published:** 2015-11-10

**Authors:** Wenqin Xiao, Weiliang Jiang, Jie Shen, Guojian Yin, Yuting Fan, Deqing Wu, Lei Qiu, Ge Yu, Miao Xing, Guoyong Hu, Xingpeng Wang, Rong Wan

**Affiliations:** 1 Department of Gastroenterology, Shanghai Tenth People’s Hospital, Tongji University School of Medicine, Shanghai, China; 2 Department of Gastroenterology, Shanghai First People’s Hospital, Shanghai Jiaotong University School of Medicine, Shanghai, China; 3 Department of Gastroenterology, Shanghai Changzheng Hospital, Shanghai Second Military Medical University, Shanghai, China; Centro Nacional de Investigaciones Oncológicas (CNIO), SPAIN

## Abstract

Pancreatic fibrosis, a prominent feature of chronic pancreatitis (CP), induces persistent and permanent damage in the pancreas. Pancreatic stellate cells (PSCs) provide a major source of extracellular matrix (ECM) deposition during pancreatic injury, and persistent activation of PSCs plays a vital role in the progression of pancreatic fibrosis. Retinoic acid (RA), a retinoid, has a broad range of biological functions, including regulation of cell differentiation and proliferation, attenuating progressive fibrosis of multiple organs. In the present study, we investigated the effects of RA on fibrosis in experimental CP and cultured PSCs. CP was induced in mice by repetitive cerulein injection *in vivo*, and mouse PSCs were isolated and activated *in vitro*. Suppression of pancreatic fibrosis upon administration of RA was confirmed based on reduction of histological damage, α-smooth muscle actin (α-SMA) expression and mRNA levels of β-catenin, platelet-derived growth factor (PDGF)-Rβ transforming growth factor (TGF)-βRII and collagen 1α1 *in vivo*. Wnt 2 and β-catenin protein levels were markedly down-regulated, while Axin 2 expression level was up-regulated in the presence of RA, both *in vivo* and *in vitro*. Nuclear translation of β-catenin was significantly decreased following RA treatment, compared with cerulein-induced CP in mice and activated PSCs. Furthermore, RA induced significant PSC apoptosis, inhibited proliferation, suppressed TCF/LEF-dependent transcriptional activity and ECM production of PSC via down-regulation of TGFβRII, PDGFRβ and collagen 1α1 *in vitro*. These results indicate a critical role of the Wnt/β-catenin signaling pathway in RA-induced effects on CP and PSC regulation and support the potential of RA as a suppressor of pancreatic fibrosis in mice.

## Introduction

Pancreatic fibrosis is a characteristic histological feature of CP and pancreatic cancer [1]. Accumulating evidence has demonstrated persistent activation of PSCs, which play a vital role in pancreatic fibrogenesis [2–5]. PSCs, which accounting for 4 to 7% of all parenchymal cells, localize in the periacinar region of exocrine pancreas [6] and store retinoids in fat droplets, similar to hepatic stellate cells (HSCs) [7]. In normal pancreas, PSCs are quiescent and can be identified by the presence of vitamin A-containing fat droplets in the cytoplasm. In response to pancreatic inflammation or injury, PSCs tend to lose their fat droplets and express α-SMA and ECM proteins as a prerequisite for activation [8–9]. Restoration of quiescence in activated PSCs has been proposed as a potential novel therapeutic strategy [10]. Quiescent PSCs can store retinol, a characteristic that is lost upon activation [10–12]. A variety of pro-fibrotic and pro-inflammatory mediators released during the development of pancreatitis, such as mitogen PDGF, the autocrine stimulator of ECM synthesis, TGF-β1 and angiotensin II, act as key cytokines promoting PSC activation. PDGF induces PSC proliferation, resulting in up-regulated migratory potential. Furthermore, the expression of α-SMA and ECM protein can be induced in PSCs by TGF-β [2,13–14], which is one of the pivotal activators involved in the majority of fibrotic conditions, including pancreatic fibrosis, hepatic fibrosis, and pulmonary fibrosis [15]. Pancreatic acinar-to-ductal metaplasia (ADM), an important hallmark of CP, as the initiating event of pancreatic intraepithelial neoplasia or pancreatic ductal adenocarcinoma, is characterized by presence of the acinar marker Amylase and the ductal marker CK19 [16].

Wnt proteins involve a family of 19 highly conserved, secreted cysteine-rich glycoproteins that play a significant role in the regulation of diverse processes, including cell survival, proliferation, migration and polarity, specification of cell fate and self-renewal of stem cells [17]. Activation of Wnt proteins involves at least four distinct intracellular signaling cascades: Wnt/β-catenin, Wnt/Ca^2+^, Wnt/planar cell polarity and Wnt/protein kinase A pathways [18]. Dysregulation of Wnt signaling leads to developmental defects and human diseases affecting either tissue development or homeostasis. The extensively characterized canonical Wnt/β-catenin signaling through the β-catenin protein to drive activation of specific target genes and regulate a diverse series of biological processes [19]. During activation of this signaling pathway, cytoplasmic β-catenin is stabilized and enters the nucleus where it associates with transcription factors, notably T cell factor and lymphoid enhancer-binding factor(TCF/LEF), to regulate target gene transcription [20–21], including myc and Axin 2 [22]. Axin 2 has been identified as a member of the destruction complex, and it functions to regulate the level of nuclear β-catenin in a negative-feedback loop, thereby being a negative regulator and target gene [23]. Rennoll and his team have found that nuclear Axin 2 represses the expression of Wnt/β-catenin-responsive luciferase reporters and forms a complex with β-catenin and TCF [22].While the non-canonical Wnt pathway is defined as Wnt signaling independent of β-catenin transcriptional function [24]. Recent research has demonstrated that Wnt/β-catenin signaling plays a vital role in the development of fibrosis in multiple organs, particularly liver [25–30]. Our group previously suggested that this pathway mediates PSC activation by up-regulating the protein levels of Wnt 2 and β-catenin, and increasing the expression of nuclear β-catenin [31].

Despite increasing research on pancreatic fibrosis, the molecular mechanisms remain largely unknown, mainly because of the unavailability of histological samples *in vivo* and lack of appropriate models *in vitro*. Progressive fibrosis of pancreas eventually leads to loss of pancreatic function and systemic complications, including malabsorption, hypercalcemia, diabetes mellitus and tumor desmoplasia [32]. Effective therapeutic procedures to treat CP remain an urgent unmet medical need. RA has a wide range of biological functions, such as regulating differentiation and proliferation of various cells [33]. Furthermore, RA treatment of pancreatic ductal cancer cells results in a less malignant phenotype and inhibits the acinar differentiation in the developing pancreas [34]. The compound has been shown to prevent the progressive fibrosis of important organs, including liver, heart, lung and kidney [35–38]. In addition, RA also can inhibit the activation of PSCs [39]. Many factors have been implicated in the pathogenesis of pancreatic fibrosis. The loss of fat droplets of PSCs has been observed over the progression of pancreatic inflammation or injury, the group of Froeling reported relative deficiency of fat-soluble vitamins, such as vitamin A, in patients with pancreatic cancer, thus perpetuating the vicious cycle of PSC activation. His team further showed that RA-induced PSC quiescence involves reduction of paracrine Wnt/β-catenin signaling pathway to slow tumor progression [10]. Several studies to date have reported that RA affects the pathophysiology of progressive fibrosis and chronic dysfunction and modulates osteogenesis and adipogenesis via Wnt/β-catenin signaling pathway, further supporting its utility as an effective therapeutic option for chronic disease [40–41].

Here, we examined the hypothesis that RA can supplement loss of fat droplets during injury of the pancreas, ameliorate progression of pancreatic fibrosis in experimental CP, and inhibit PSC activation via suppressing the Wnt/β-catenin signaling pathway. The effects of RA were investigated both *in vivo* and *in vitro* using a mouse model of cerulein-induced CP and a cellular model of mouse PSC.

## Materials and Methods

### Ethics statement

All animal-related procedures were approved by the Animal Care and Use Committee of The Tenth People’s Hospital of Shanghai, Tongji University. This research was also approved by the Science and Technology Commission of Shanghai Municipality (ID: SYXK 2007–0006) under the permit number 2011-RES1. Mice were maintained under a 12 h light–dark cycle at 22°C, provided water *ad libitum*, fed standard laboratory chow, and allowed to acclimatize for a minimum of one week. The environment was maintained at a relative humidity of 30–70%. Mice with cerulein-induced pancreatitis were monitored as described above.

### Animals and experimental design

**Fig 1:**
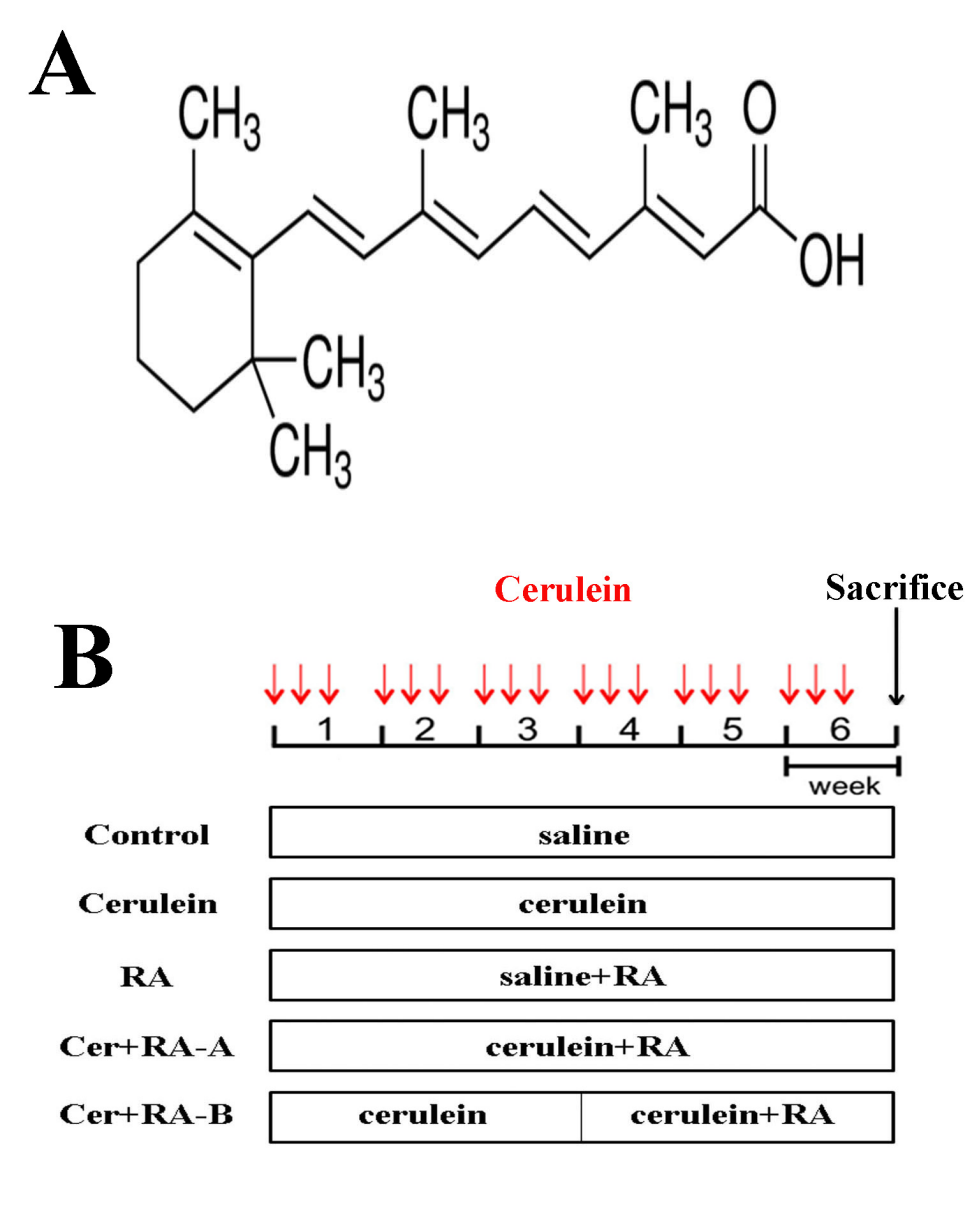
Compounds used and schematic chart of the experimental design. **(A)** Chemical structure of retinoic acid, a retinoid, with a low molecular weight of 300.4. **(B)** Five groups of mice (n = 8) were analyzed. The Control group received 6 injections of saline only whereas the Cerulein group received 6 injections of cerulein at a dose of 50 μg/kg 3 days a week. The RA group was administered 6 injections of saline and RA (20 mg/kg) 3 days a week. The Cer+RA-A group was given 6 injections of cerulein and RA from the first day of the experimental period for a total of 6 weeks whereas the Cer+RA-B group received the same cerulein injections as the Cer+RA-A group, but administered RA from the first day of week 4 until the end of the experiment over a total period of 3 weeks.

Male Balb/c mice were purchased from Shanghai SLAC Laboratory Animal Co., Ltd. (Shanghai, China). Mice (n = 40) weighing 20 ± 2 g were randomly assigned to 5 groups of 8 animals each. CP injury and pancreatic fibrosis were induced in mice via repetitive acute pancreatitis episodes [42]. Acute pancreatitis was induced via hourly (6 times) intraperitoneal injections of 50 μg/kg body weight cerulein, meanwhile control animals received a comparable amount of normal saline (NS) [43]. Cerulein group received 6 intraperitoneal (i.p.) injections of cerulein (Sigma-Aldrich, St.Louis, MO, USA) at the dose of 50 μg/kg 3 days a week and 0.1 mL fat emulsion after 6 injections of cerulein while control animals received a comparable amount of NS and 0.1 mL fat emulsion after 6 injections. The RA group was administered NS injections and RA (98% purity, Sigma-Aldrich, St.Louis, MO, USA) dissolved in 0.1 mL fat emulsion at 20 mg/kg (Fig 1A). The Cer+RA-A group received cerulein injections along with 20 mg/kg RA 3 days a week from the first day of the experimental period for a total of 6 weeks, mimicking a preventive course of treatment. The Cer+RA-B group received cerulein injections similar to the Cer+RA-A group, but RA treatment started from the first day of week 4 until the end of experiment for a total of 3 weeks, mimicking a therapeutic course of treatment. Mice were sacrificed at the end of the 6-week study period under anesthesia with 3% pentobarbital sodium (Fig 1B). In each case, the pancreas was rapidly removed, and a proportion fixed in 4% paraformaldehyde buffered with phosphate-buffered saline (PBS) overnight at 4°C and embedded in paraffin wax or immediately frozen at −80°C. The remaining fraction was quickly ground into liquid nitrogen and frozen at −80°C for further study. Blood samples were maintained at room temperature for 2 h before centrifugation for 3000 *g* at 4°C for 15 min, and serum stored at −80°C.

### Serum amylase and lipase analyses

Serum levels of amylase and lipase were measured via enzyme dynamics chemistry using commercial kits on a Roche/Hitachi modular analytics system (Roche, Mannheim, Germany), according to the manufacturer’s protocol.

### Histological examination

Mouse pancreas histology was examined via hematoxylin-eosin (H&E) and Masson’s trichrome staining. A proportion of pancreatic tissue was fixed in 4% phosphate-buffered formaldehyde in 24 h, dehydrated via a graduated ethanol series, and embedded in paraffin blocks. Pancreas sections (5 μm) were dewaxed in xylene, hydrated through an upgraded ethanol series, and subjected to H&E and Masson trichrome staining. Morphological changes were examined under a light microscope by three pathologists who were unaware of the origin of the specimens. In brief, the severity of CP was assessed using a semiquantitative graded score: graded glandular atrophy (0–3), intralobular, interlobular and periductal fibrosis (0–3) and inflammatory mononuclear infiltrates (0–3) [44].

### Immunohistochemical study of α-SMA and β-catenin

Formalin-fixed, paraffin-embedded samples were cut into 5 μm thick sections. Tissue sections were deparaffinized and rehydrated with upgraded ethanol. For antigen retrieval, slides were boiled in EDTA (1 mmol/L, pH 8.0) for 15 min in a microwave oven. Endogenous peroxidase activity was quenched with 0.3% hydrogen peroxide solution for 10 min at room temperature. After rinsing with PBS, slides were blocked with bovine serum albumin (BSA) in PBS for 30 min. Sections were subsequently incubated with mouse monoclonal α-SMA (1:800 dilution, Santa Cruz, California, USA) and rabbit polyclonal β-catenin (1:800 dilution, CST, Danvers, MA, USA) antibodies overnight at 4°C. Antibody binding was detected with an Envision Detection Kit, Peroxidase/DAB, Rabbit/Mouse (Gene Tech, Shanghai, China). Next, sections were counterstained with hematoxylin. As a negative control, PBS replaced the primary antibody. Areas staining positive for α-SMA and β-catenin were observed in all specimens under a microscope by three pathologists who were unaware of the specimen origins (CTR 6000; Leica, Wetzlar, Germany).

### Cell culture and Retinoic Acid treatment

PSCs were isolated from male Balb/c mice via digestion of pancreatic tissue and Nycodenz density gradient centrifugation, as described previously [45–46]. Freshly isolated mice PSCs were cultured in DMEM/F12 (Gibco BRL, USA) supplemented with 10% fetal bovine serum (FBS; Gibco BRL, USA) and 1% penicillin–streptomycin (PS; Gibco BRL, USA) at 37°C, 5% CO_2_. The next day and every 2 days thereafter, the culture medium was changed and treated with different doses of RA (0, 0.5, 1, 2 μmol/L) or left untreated. Murine pancreatic acinar cell line 266–6 (Cobioer ATCC, USA) was cultured in DMEM/HIGH GLUCOSE(Gibco BRL, USA) supplemented with 10% FBS and 1% PS at 37°C, 5% CO_2_, the culture medium was changed every 2 days and administrated with or without 100 nmol/L cerulein for 5 days [16], then treated with or without 1 μmol/L RA.

### Immunofluorescence staining

Formalin-fixed, paraffin-embedded samples were prepared as described above and incubated overnight at 4°C with a primary goat monoclonal antibody Ki-67 (1:100 dilution, Santa Cruz, California, USA), and α-SMA (1:400 dilution). Freshly isolated mouse PSCs were cultured as described previously. On day 5, cells were collected, washed in PBS (3×5min), and fixed in 4% paraformaldehyde for 20 min. After washing in PBS (3×5min), cells were blocked for 20 min with 5% BSA and incubated overnight at 4°C with Ki-67(1:100 dilution) and β-catenin antibody (1:200 dilution). The next day, cells and tissue sections were washed in PBS (3×5min). Cells followed by immunofluorescence detection using a donkey anti-goat antibody (1:400 dilution) conjugated with fluorochrome Cy3 (Jackson ImmunoResearch Laboratory, USA) and donkey anti-rabbit antibody (1:200 dilution) conjugated with fluorochrome Alexa FluorH488 (Jackson ImmunoResearch Laboratory, USA), and tissue sections using a donkey anti-goat antibody (1:400 dilution) conjugated with fluorochrome Cy3 and donkey anti-mouse antibody (1:200 dilution) conjugated with fluorochrome Alexa FluorH488 for 1 h in the dark at 37°C. After washing in PBS (3×5min), cells and tissue sections were mounted in Fluoromount^TM^ mounting medium (Sigma-Aldrich, St. Louis, Missouri, USA) with 49, 6-diamidino-2-phenylindole (DAPI) (1:1000 dilution, Sigma-Aldrich, St. Louis, MO, USA). Fluorescence analysis was performed using a confocal laser scanning microscope (LSM710; Zeiss, Germany) and Zen 2011 software (Carl-Zeiss, Jena, Germany).

### Real-Time Quantitative PCR

Total RNA was extracted from pancreas, mouse PSCs and 266–6 cells using TRIzol reagent (Invitrogen, Carlsbad, CA, USA) following the manufacturer’s instructions, and subjected to reverse transcription using the PrimeScript RT reagent Kit (TaKaRa, Japan). Quantitative real-time PCR (qRT-PCR) was performed in triplicate for each gene of interest using the ABI Prism 7900 HT Sequence Detection System (Applied Biosystems, Carlsbad, CA, USA), according to the SYBR Premix EX Taq manual (TaKaRa). GAPDH was used as a separate endogenous control to which the gene of interest was normalized, and fold changes for gene expression calculated using the comparative CT (2−ΔΔCT) method. Primer sequences for biomarkers were designed with software are shown in Table 1.

**Table 1:** Primer Sequences Used for qRT-PCR Analysis.

Gene		Primer sequence (5’→3’)
Amylase	Forward	CAAAATGGTTCTCCCAAGGA
	Reverse	ACATCTTCTCGCCATTCCAC
CK19	Forward	ACCCTCCCGAGATTACAACC
	Reverse	CAAGGCGTGTTCTGTCTCAA
α-SMA	Forward	TGCCGAGCGTGAGATTGT
	Reverse	CCCGTCAGGCAGTTCGTAG
β-catenin	Forward	AGGGTGCTATTCCACGACTA
	Reverse	CACCCTTCTACTATCTCCTCCAT
Axin 2	Forward	GTCTCTACCTCATTTCCCGAGAAC
	Reverse	CGAGATCAGCTCAGCTGCAA
PDGFRβ	Forward	CCAGAAGTAGCGAGAAGC
	Reverse	ATCACCGTATCGGCAGTA
TGFβRII	Forward	TTTCGGAAGAATACACCAC
	Reverse	GACACGGTAGCAGTAGAA
collagen1α1	Forward	CGCCATCAAGGTCTACTG
	Reverse	ACGGGAATCCATCGGTC
GAPDH	Forward	GGTCGGTGTGAACGGATTTG
	Reverse	TGTAGACCATGTAGTTGAGGTCA

### Western blot analysis

For Western blot, mouse pancreatic tissues were retrieved from storage and rapidly ground in liquid nitrogen. The resulting powder or PSCs were lysed using a Nuclear and Cytoplasmic Protein Extraction kit (Beyotime) following the manufacturer’s protocol (Pierce, CA, USA). Whole proteins of pancreatic tissues or PSCs were reconstituted in ice-cold RIPA buffer containing phenylmethanesulfonyl fluoride (PMSF, 1 mmol/L) and a cocktail of protease inhibitors (1:100 dilution; Sigma-Aldrich), and homogenates centrifuged at 12000 *g* for 15 min at 4°C. Protein concentrations were determined using the BCA method (Beyotime). Equal amounts of protein were electrophoresed using sodium dodecyl sulfate/polyacrylamide gel electrophoresis (SDS-PAGE Bio-Rad, CA, USA) and transferred electrophoretically to membranes following the standard method. Non-specific binding was blocked with 5% low-fat milk at room temperature for 1 h in a covered container. Membranes were incubated overnight at 4°C with anti-α-SMA (1:400 dilution), anti-β-catenin (1:1000 dilution), anti-Wnt 2 (1:1000 dilution, Epitomics, Burlingame, CA, USA), anti-Axin 2 (1:1000 dilution, Abcam, Cambridge, MA, USA), anti-Lamin A (1:1000 dilution, Abcam, Cambridge, MA, USA), anti-β-actin (1:1000 dilution, Sigma-Aldrich, St.Louis, MO, USA), and anti-GAPDH (1:500 dilution, Epitomics, Burlingame, CA, USA). The next day, membranes were washed and incubated with a secondary goat anti-rabbit IgG-horseradish peroxidase (HRP) antibody (1:2000) and goat anti-mouse IgG-HRP antibody (1:2000) (Santa Cruz Biotechnology, CA, USA) for 1 h at room temperature. Finally, membranes were washed and developed using the ECL detection system (Santa Cruz Biotechnology, Santa Cruz, CA, USA).

### Cell proliferation

Freshly isolated mouse PSCs were seeded at a density of 2×10^5^ cells per well in 24-well plates and cultured as described previously. After 24 h, cells were treated as indicated and incubated for 3 days. Proliferation was determined using Cell Counting Kit-8 (CCK-8, Sigma-Aldrich, St. Louis, MO, USA) according to the manufacturer's instructions. For the BrdU incorporation assay, 30 mmol/L BrdU (Sigma-Aldrich, St. Louis, MO, USA) was added to culture medium for incorporation into DNA of replicating cells. After 2 h of incubation, cells were fixed in 4% paraformaldehyde and immunostained for BrdU using the manufacturer's protocol. Finally, proliferative cells were counted in 10 different fields at × 200 magnification in three independent experiments by three professionals.

### Cell apoptosis

Freshly isolated mouse PSCs were seeded at a density of 2×10^6^ cells in 25 cm^2^ coated plastic bottles and cultured as described previously. On day 5, cells were collected, washed in PBS (3×5min), mixed in 100 μL 1× binding buffer, and incubated at room temperature for 15 min with an Annexin-V/PI (BD Biosciences) double staining solution. Stained cells were detected using flow cytometry and the percentage of apoptotic cells calculated using ModFitLT software (Verity Software House). Apoptosis in mouse PSCs was analyzed using Hoechst 33342 staining (Beyotime). Briefly, cells were stained with 10 mg/ml Hoechst 33342 and examined directly using fl. Briefly, microscopy. Cells with nuclei containing condensed and/or fragmented chromatin were considered apoptotic [47]. Viable (Hoechst negative/dark blue) and apoptotic (Hoechst positive/light blue) cells were counted in 10 different fields at × 200 magnification in each well in three independent experiments by three professionals.

### Dual-luciferase assay

The luciferase assay of TOPflash/FOPflash is widely used to evaluate the.transcriptional activity of β-catenin/TCF dependent signaling [48–49]. PSCs were seeded at a density of 1×10^5^ cells per well in 24-well plates and transfected with 0.2 μg of TOPflash (a TCF/LEF-responsive reporter) or FOPflash (a negative control) reporter gene plasmids, and 0.01 μg of pRL-SV40 (Promega, USA) using Lipofectamine LTX/Plus Reagent (Invitrogen, USA). Then we performed our co-transfection experiments, cells were transfected with 0.2 μg of S33Y-β-catenin (a stabilized mutant β-catenin) plasmid or a control empty plasmid plus the reporter plasmid. After 12 h of incubation, we added different doses of RA (0.5, 1 and 2 μmol/L) into each well. After 72 h, we evaluated the luciferase activity as described previously [48]. TCF/LEF-dependent transcriptional activity was normalized to Renilla luciferase activity from the control plasmid pRL-SV40.

### Statistical analysis

Results were expressed as mean ± standard deviation (SD). Statistical analysis was performed using one-way ANOVA, followed by Student–Newman–Keuls (SNK) as a post hoc test. The Kruskal-Wallis test was used to evaluate differences in categorical values, followed by the Mann-Whitney U post hoc test. Data were considered statistically significant at p < 0.05.

## Results

### Effects of RA on serum amylase, lipase, body weight and organs

**Fig 2:**
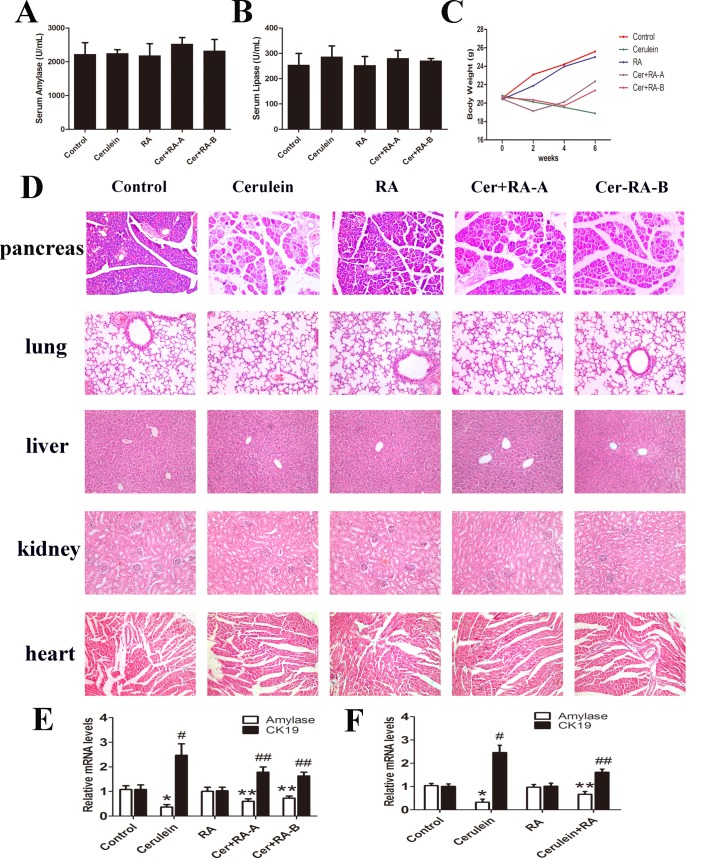
Assessment of chronic pancreatitis in mice. **(A and B)** Activities of serume amylase and lipase were analyzed. No obvious changes were evident among all groups. **(C)** Assessment of body weight among the 5 groups of mice in the 6-week experimental period revealed no changes between Control and RA groups. **(D)** Pancreatic tissues in all groups were evaluated via H&E staining. Control and RA groups showed normal pancreatic histological features and severe pancreatic damage was observed in the Cerulein group. Cer+RA-A and Cer+RA-B groups showed less pancreatic damage. Tissues of lung, liver, kidney and heart in all groups analyzed via H&E staining remained statistically unchanged among all groups (magnification: ×200). **(E and F)** Quantitative RT-PCR analysis of the mRNA levels of Amylase and CK19 in cerulein-induced CP and cerulein-administrated 266–6 cells. Results are presented as mean±SD from three independent experiments. *p < 0.05 and ^#^ *p* < 0.05, compared with the Control and RA groups, **p < 0.05 and ^##^ *p* < 0.05 compared with the Cerulein group.

The levels of serum amylase and lipase, histology of pancreatic tissue and body weight changes were similar between Control and RA groups, indicating no adverse effects of RA on these parameters (Fig 2A–2D). No significant histological changes in lung, liver, kidney and heart were evident in all groups (Fig 2D).

### RA ameliorates cerulein-induced chronic pancreatitis

Six weeks of RA administration led to no obvious histological changes in pancreatic tissue sections between Control and RA groups, indicating that long-term treatment with RA (20 mg/kg) is not toxic to pancreas. In Cerulein, Cer+RA-A and Cer+RA-B groups, histological features of CP were evident at the time of sacrifice (week 6), including abnormal architecture, glandular atrophy, pseudotubular complexes, fibrosis, and inflammatory cell infiltrates. The Cer+RA-A and Cer+RA-B groups displayed less severe pancreatic damage, based on evaluation of histological scores (Table 2 and Fig 2D). To explore whether RA has potential effect in ADM, a hallmark of CP, we detected the markers of ADM via Quantitative RT-PCR analysis both in cerulein-induced CP and cerulein-administrated 266–6 cells. Quantitative RT-PCR results showed down-regulated Amylase mRNA expression in the Cerulein group, compared with Control and RA groups, this down-regulation was markedly suppressed in both Cer+RA-A and Cer+RA-B groups. However, Cerulein had a positive effect on the mRNA expression of CK19, and administration of RA inhibited this effect in vivo (Fig 2E). The mRNA expression level of Amylase was markedly decreased in 266–6 cells administration of cerulein, compared with Control and RA groups, and this decrease was suppressed in 266–6 cells treatment of RA. However, Cerulein had a positive effect on the mRNA expression of CK19, and treatment of RA strongly inhibited this effect *in vitro* (Fig 2F).

**Table 2:** RA reduces the severity of experimental cerulein-induced CP.

	Control	Cerulein	RA	Cer+RA-A	Cer+RA-B
Glandular atrophy	0±0	2.54±0.32*	0±0	1.56±0.21^#^	1.67±0.18^#^
Fibrosis	0±0	2.35±0.28*	0±0	1.24±0.18^#^	1.47±0.22^#^
Inflammation	0±0	2.48±0.23*	0±0	1.39±0.22^#^	1.56±0.17^#^
Damage index(DI)	0±0	7.35±0.56*	0±0	3.87±0.28^#^	4.12±0.21^#^

Data are presented as mean±SD from three independent experiments.

*p < 0.05, compared with Control and RA groups

^#^ *p* < 0.05, compared with the Cerulein group.

### RA attenuates fibrosis in pancreatic tissue

**Fig 3:**
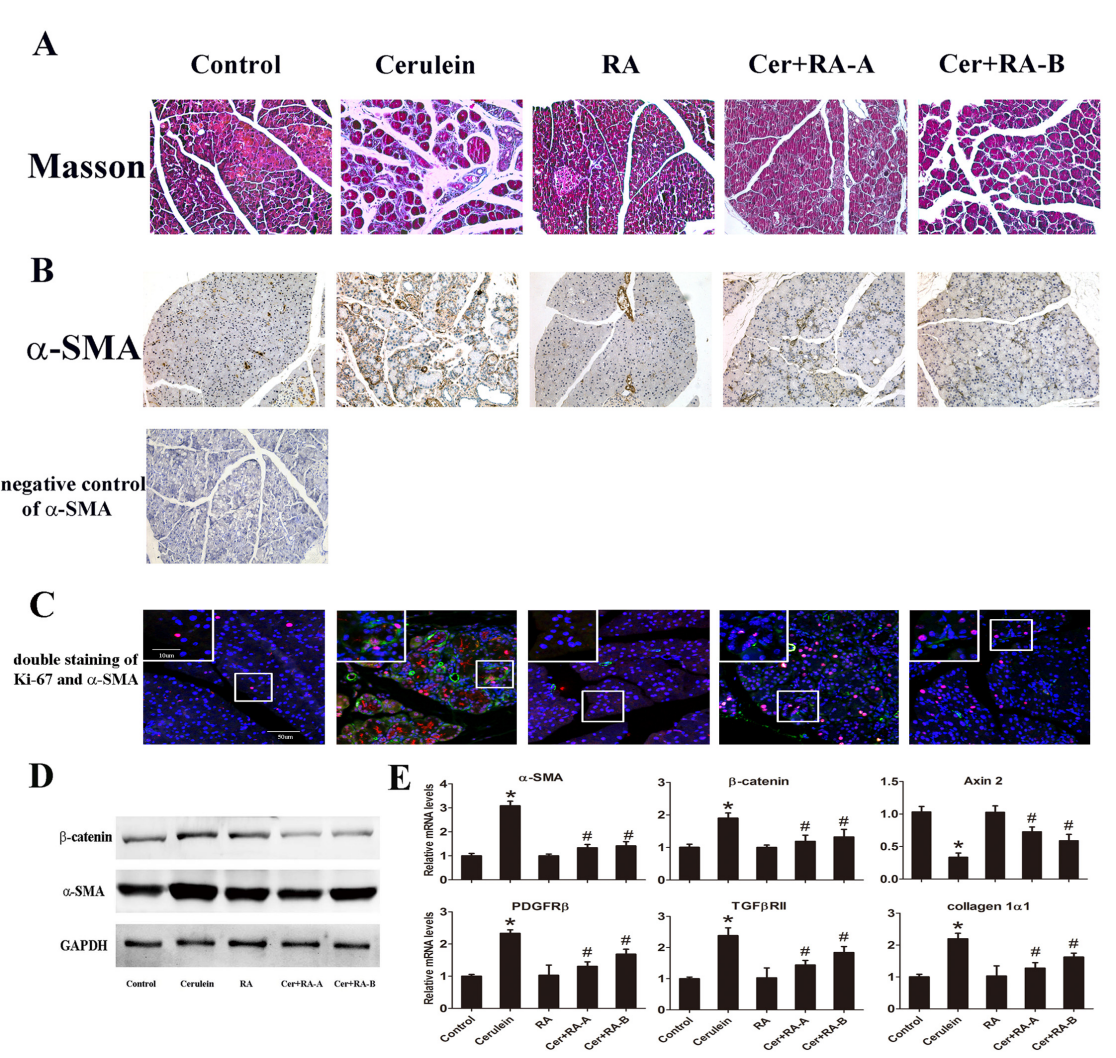
RA attenuates pancreatic fibrosis in cerulein-induced CP mice. **(A)** Pancreatic tissue sections stained with Masson (magnification: ×200). **(B)** Immunochemical staining of pancreatic tissue sections for α-SMA (magnification: ×200). **(C)** Double immunofluorescence staining of Ki-67 (red) and α-SMA (green) (magnification: ×250, inset: ×1250). **(D)** Western blot analysis of α-SMA and β-catenin protein expression in 5 groups. GAPDH was used as the internal reference. Figures are representative of three independent experiments. **(E)** Quantitative RT-PCR analysis of the mRNA levels of α-SMA, β-catenin, Axin 2, PDGFRβ, TGFβRII and collagen1α1. GAPDH was used as the housekeeping control. Data are presented as mean±SD of three independent experiments. *p < 0.05, compared with the Control and RA groups, and ^#^ *p* < 0.05 compared with the Cerulein group.

Pancreas sections stained with Masson were analyzed to evaluate the degree of fibrosis. Pancreatic collagen content was obviously increased after induction of CP, which was ameliorated upon administration of RA (Fig 3A). Areas of positive Masson-stained sections of the Cerulein group were higher, compared with the Control and RA groups, and in Cer+RA-A and Cer+RA-B groups, positive Masson-stained areas were decreased, compared with that in Cerulein group. Immunohistochemistry of α-SMA was performed to quantify the number of activated stellate cells. Expression was significantly increased in the Cerulein group, compared with Control and RA groups, and markedly reduced in Cer+RA-A and Cer+RA-B groups (Fig 3B). Double immunofluorescence staining of Ki-67 and α-SMA showed the number of activated stellate cells. Consistently, expression was increased in the Cerulein group, compared with Control and RA groups, and reduced in Cer+RA-A and Cer+RA-B groups (Fig 3C). Western blot results showed up-regulation of α-SMA and β-catenin in the Cerulein group, compared with Control and RA groups. Moreover, this up-regulation was markedly suppressed in both Cer+RA-A and Cer+RA-B groups (Fig 3D). Consistently, quantitative RT-PCR results showed an evident increase in the mRNA expression levels of α-SMA, β-catenin, TGFβRII, PDGFRβ and collagen 1α1 in the Cerulein group, compared with Control and RA groups. This up-regulation was significantly suppressed in both Cer+RA-A and Cer+RA-B groups (Fig 3E). Axin 2 is a direct Wnt/β-catenin target gene, and can negatively regulate Wnt/β-catenin signaling pathway [23], which prompted us to investigate whether Axin 2 expression is affected in the Cerulein-induced CP. To this end, we further performed Quantitative RT-PCR to examine the expression of Axin 2, and observed Axin 2 mRNA level was reduced in Cerulein group, compared with Control and RA groups. This reduction was greatly inhibited in both Cer+RA-A and Cer+RA-B groups (Fig 3E).

### RA modulates the Wnt/β-catenin signaling pathway via inhibiting nuclear translocation of β-catenin in chronic pancreatitis

**Fig 4:**
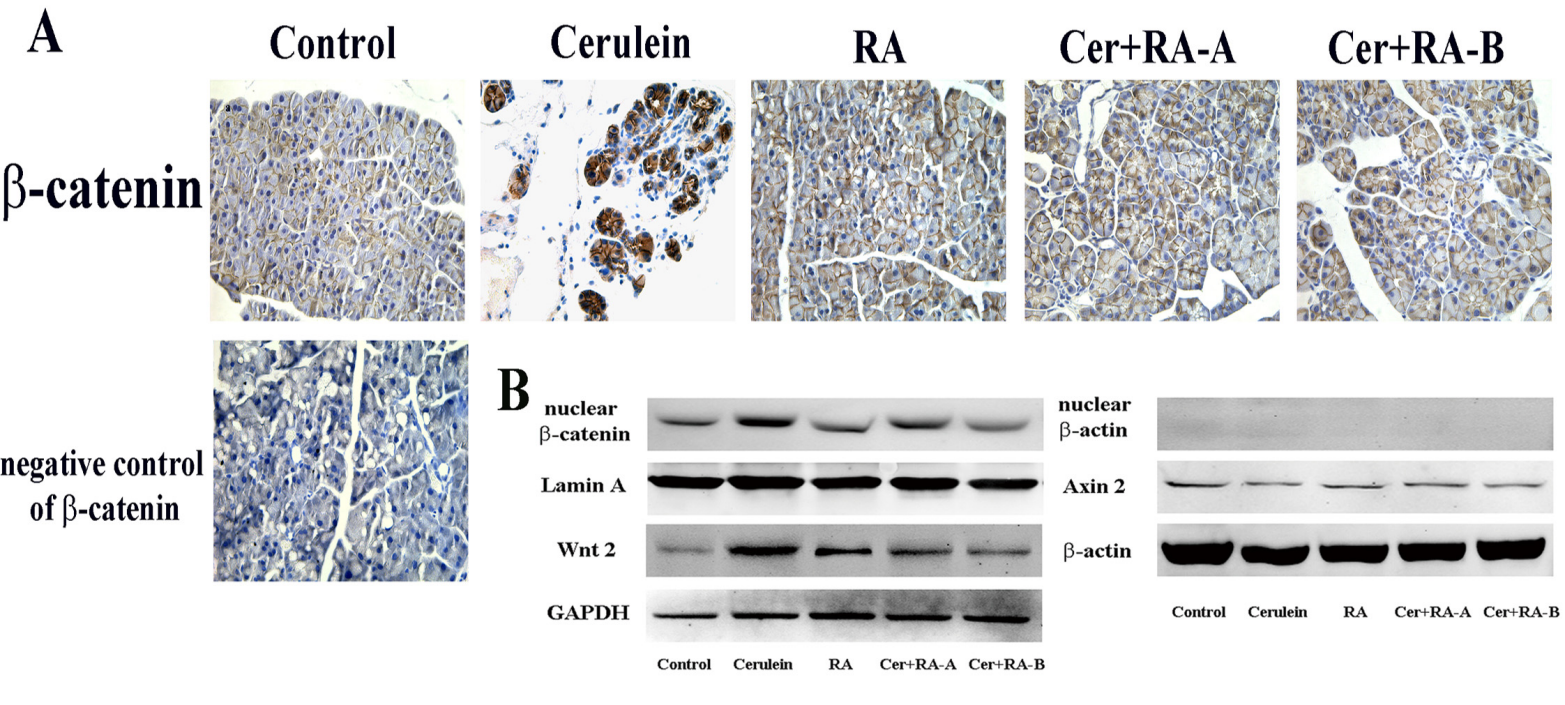
RA inhibits nuclear translocation of β-catenin in cerulein-induced CP. **(A)** Immunohistochemical staining of β-catenin to detect nuclear translocation in CP (magnification: ×400). **(B)** Western blot analysis of the protein levels of Wnt 2, Axin 2 and nuclear β-catenin. β-actin, GAPDH and Lamin A were used as the internal references. Results are representative of three independent experiments.

To determine whether RA affects β-catenin expression, we examined the translocation of β-catenin via immunofluorescence staining and Western blot analysis. Immunofluorescence staining results showed an evident increase in nuclear translocation of β-catenin in the Cerulein group, compared with the Control and RA groups, which was significantly inhibited in the Cer+RA-A and Cer+RA-B groups (Fig 4A). In Western blot experiments, protein levels of Wnt 2 and nuclear β-catenin were up-regulated in the Cerulein group, compared with Control and RA groups. This increase in protein expression was markedly suppressed in the Cer+RA-A and Cer+RA-B groups (Fig 4B). However, Cerulein had a positive effect on the protein expression of Axin 2, and administration of RA inhibited this effect (Fig 4B).

### RA inhibits PSC proliferation and promotes apoptosis *in vitro*

**Fig 5:**
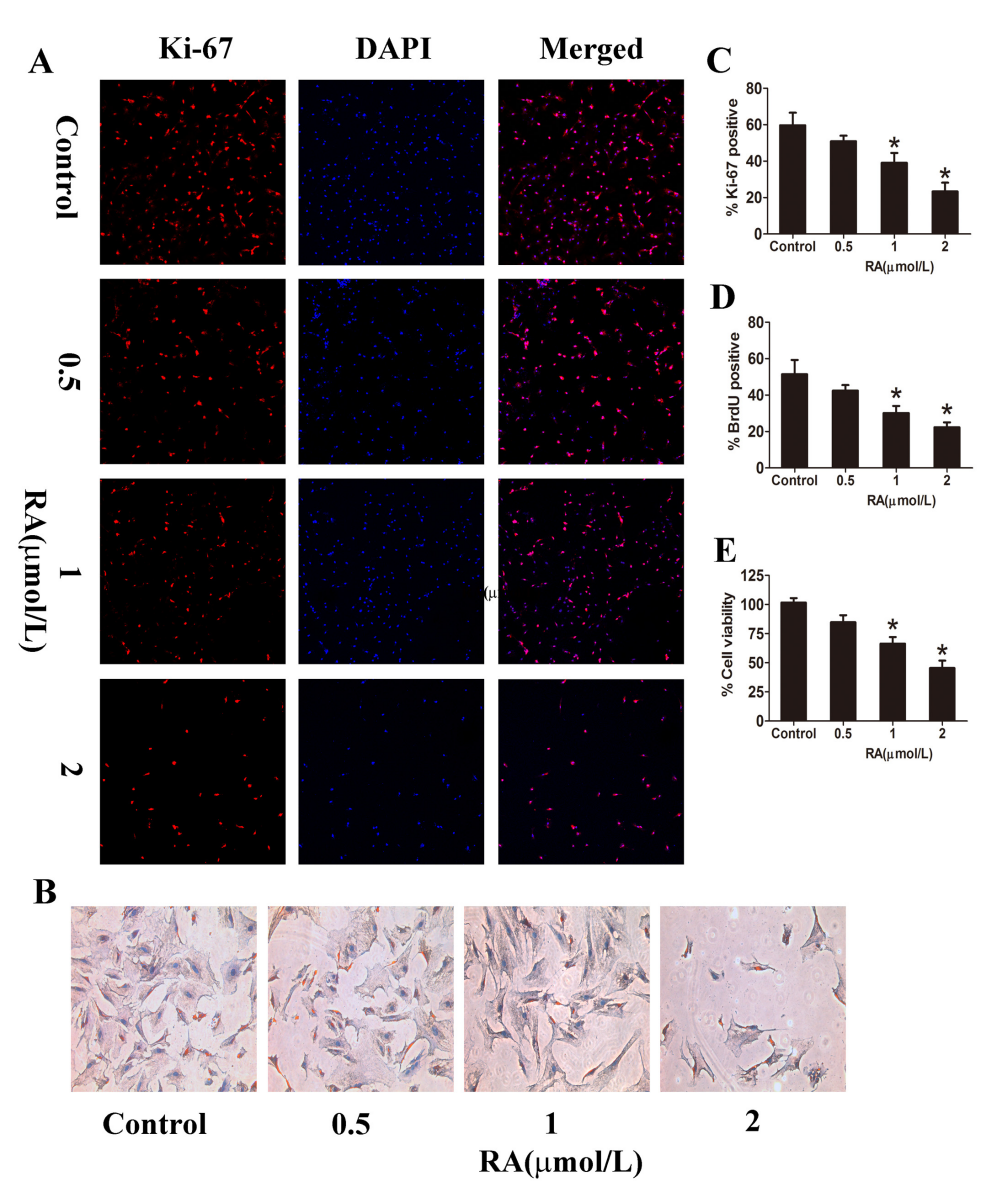
RA inhibits proliferation of PSCs *in vitro*. **(A)** Double immunofluorescence staining of DAPI (blue) and Ki-67 (red) indicated overlapping distribution patterns, confirming the anti-proliferative effect of RA (magnification: ×100). **(B)** BrdU staining of PSCs (magnification: ×200). **(C and D)** Analysis of Ki-67 and BrdU staining. **(E)** Day 1 PSCs were cultured with different doses of RA (0, 0.5, 1, 2 μmol/L) for 72 h, and cell viability detected with CCK-8. Data are presented as mean±SD from three independent experiments. *p < 0.05, compared with the Control group.

**Fig 6:**
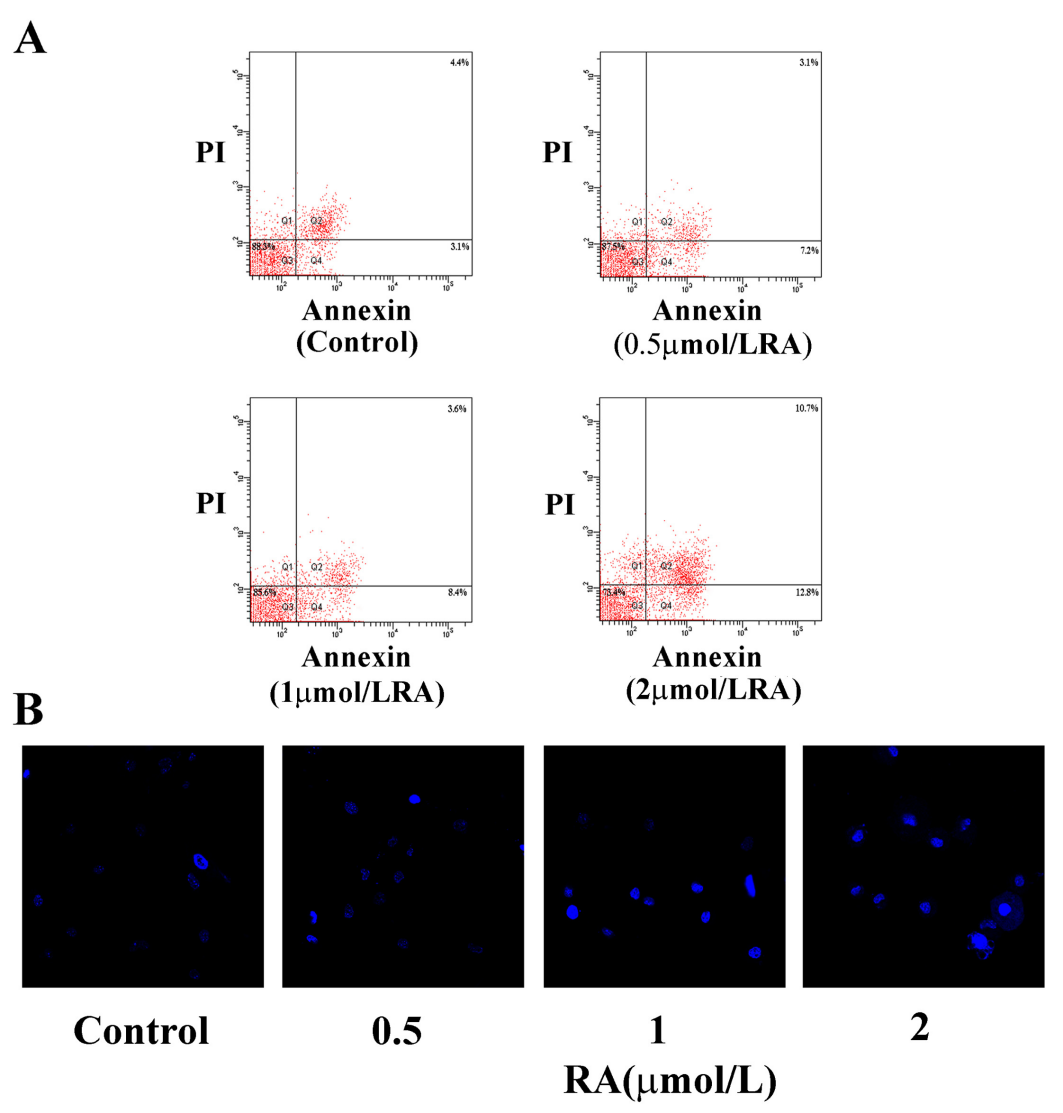
RA promotes apoptosis of PSCs *in vitro*. **(A)** PSCs were incubated with Annexin V for 15 min at room temperature in combination with PI (5 μg/ml). The rates of apoptosis were compared among PSCs treated with or without RA at different doses (0, 0.5, 1, 2 μmol/L) using flow cytometry. **(B)** Hoechst 33342 staining (magnification: ×630). Data are presented as mean±SD from three independent experiments. *p < 0.05, compared with the Control group.

Day 1 PSCs were incubated with or without RA at different doses (0, 0.5, 1, 2 μmol/L) as described previously. Day 5 PSCs were collected and cell proliferation analyzed with CCK-8, a sensitive colorimetric assay. As presented in Fig 5E, addition of RA suppressed PSC proliferation. Interestingly, we observed a dose-dependent manner in this effect. RA-induced inhibition became significant at the dose of 1 μmol/L, and even more dramatically at the dose of 2 μmol/L. Analysis of Ki-67 expression in PSC nuclei indicated an obvious reduction in the proliferation rate upon exposure to RA (Fig 5A and 5C). Furthermore, BrdU incorporation was significantly decreased in PSCs supplemented with RA, compared with control PSCs (Fig 5B and 5D). In addition, we investigated the effects of RA on PSC apoptosis via Annexin-V/PI staining. Treatment with RA induced a marked increase in the rate of apoptosis (Fig 6A). Quantification of cell apoptosis with Hoechst 33342 further confirmed induction of apoptosis by RA (Fig 6B). These findings collectively demonstrated that RA significantly inhibits the proliferation, while promotes the apoptosis of PSCs *in vitro*.

### RA inhibits PSC activation *in vitro*

**Fig 7:**
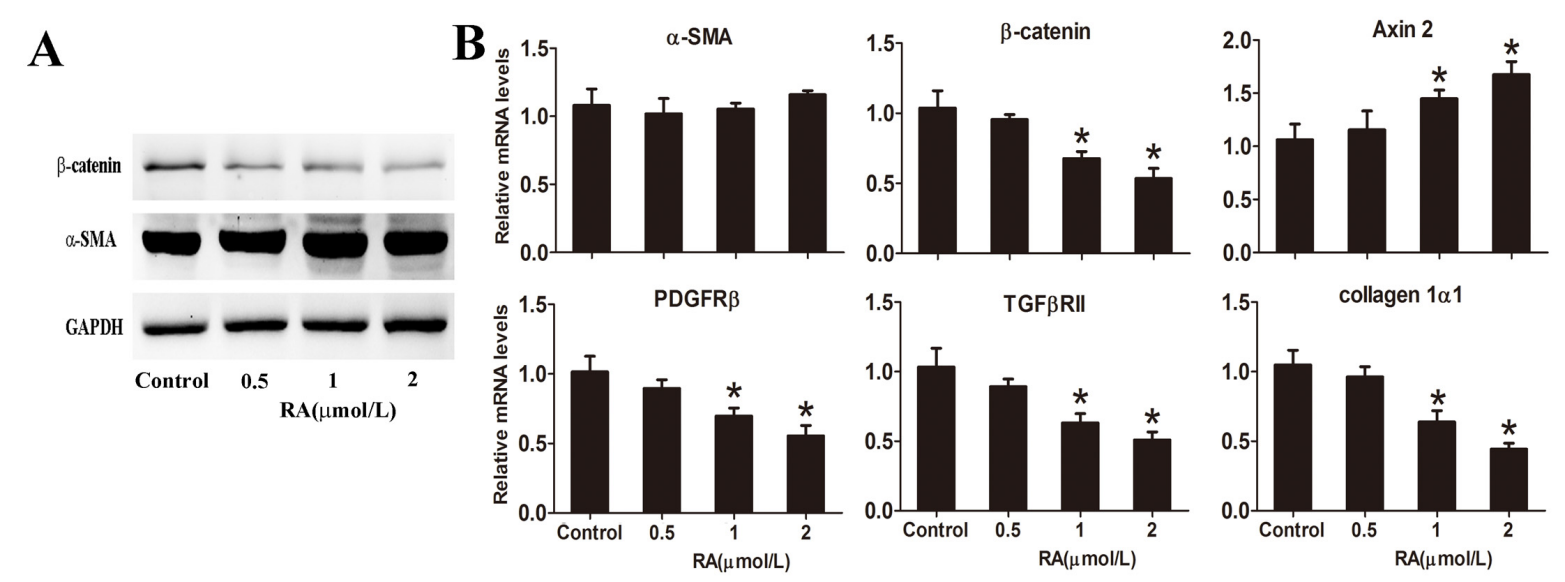
RA inhibits activation of PSCs *in vitro*. PSCs isolated from mice were cultured for 24 h and incubated with or without RA at different doses (0, 0.5, 1, 2 μmol/L). **(A)** PSCs were cultured for 5 days and protein expression levels of α-SMA and β-catenin analyzed via Western blot. GAPDH was used as the internal reference. **(B)** The mRNA levels of α-SMA, β-catenin, Axin 2, PDGFRβ, TGFβRII and collagen1α1 were detected using quantitative RT-PCR. GAPDH was used as the housekeeping control. Data are presented as mean±SD from three independent experiments. *p < 0.05 compared with the Control group.

Day 5 PSCs were collected as described previously for use in Western blot and quantitative RT-PCR analyses. RA induced marked down-regulation of β-catenin protein and mRNA expression, while Axin 2 mRNA level was up-regulated, supporting our finding that the Wnt/β-catenin pathway was partly inhibited by RA (Fig 7A and 7B). However, treatment with RA did not affect α-SMA protein and mRNA expression, compared with PSCs activated normally *in vitro* (Fig 7A and 7B). The mRNA expression levels of TGFβRII, PDGFRβ and collagen 1α1 were markedly decreased in PSCs treated with RA, compared with cells in the control group (Fig 7B).

### RA inhibits Wnt/β-catenin signaling pathway in PSCs *in vitro*

**Fig 8:**
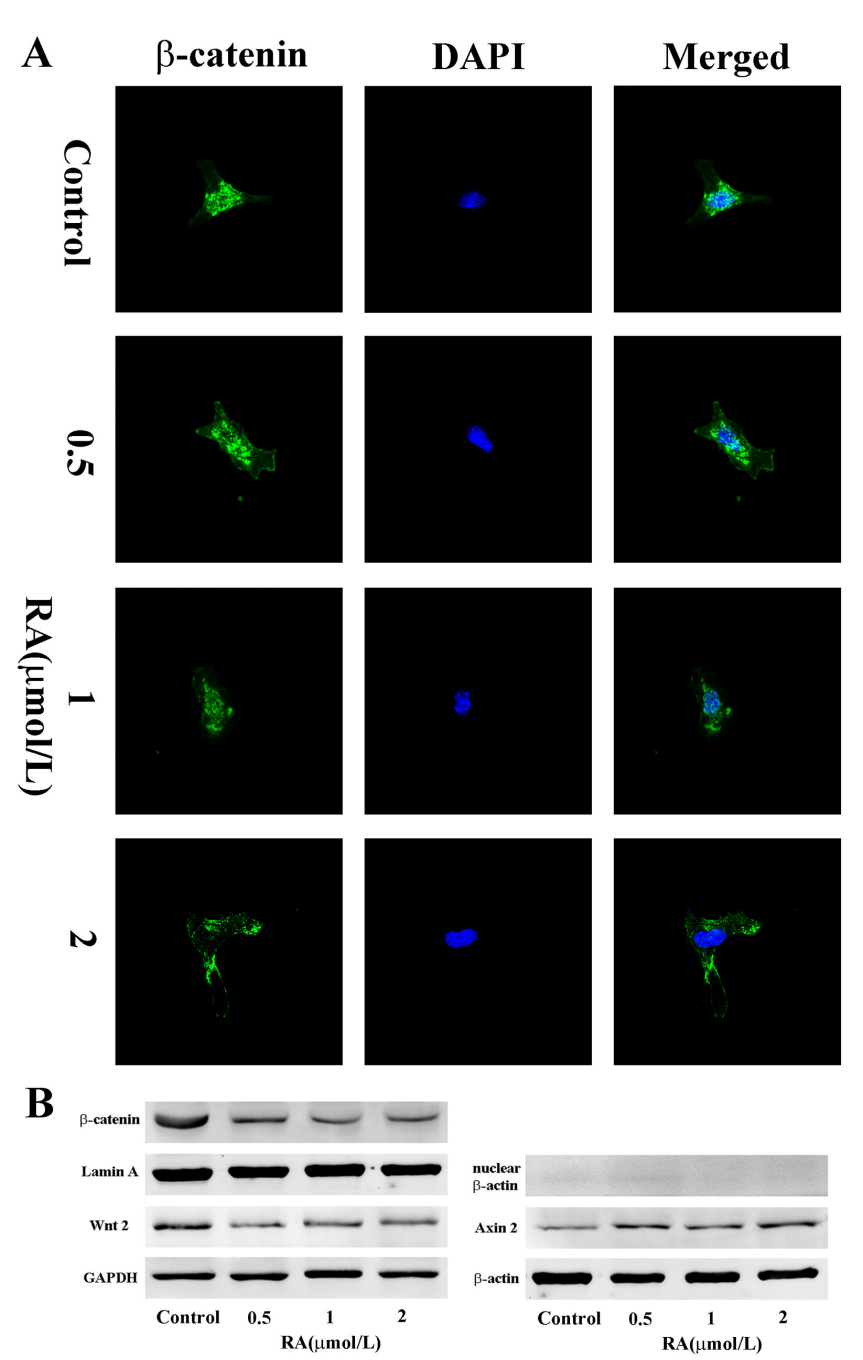
RA inhibits the nuclear translocation of β-catenin in PSCs *in vitro*. **(A)** Immunofluorescence staining of β-catenin (green) in PSCs and DAPI (blue) to counterstain nuclei (magnification: ×630). **(B)** Wnt 2, Axin 2 and nuclear β-catenin protein levels were detected via Western blot. β-actin, GAPDH and Lamin A were used as the internal references. Results are representative of three independent experiments.

**Fig 9:**
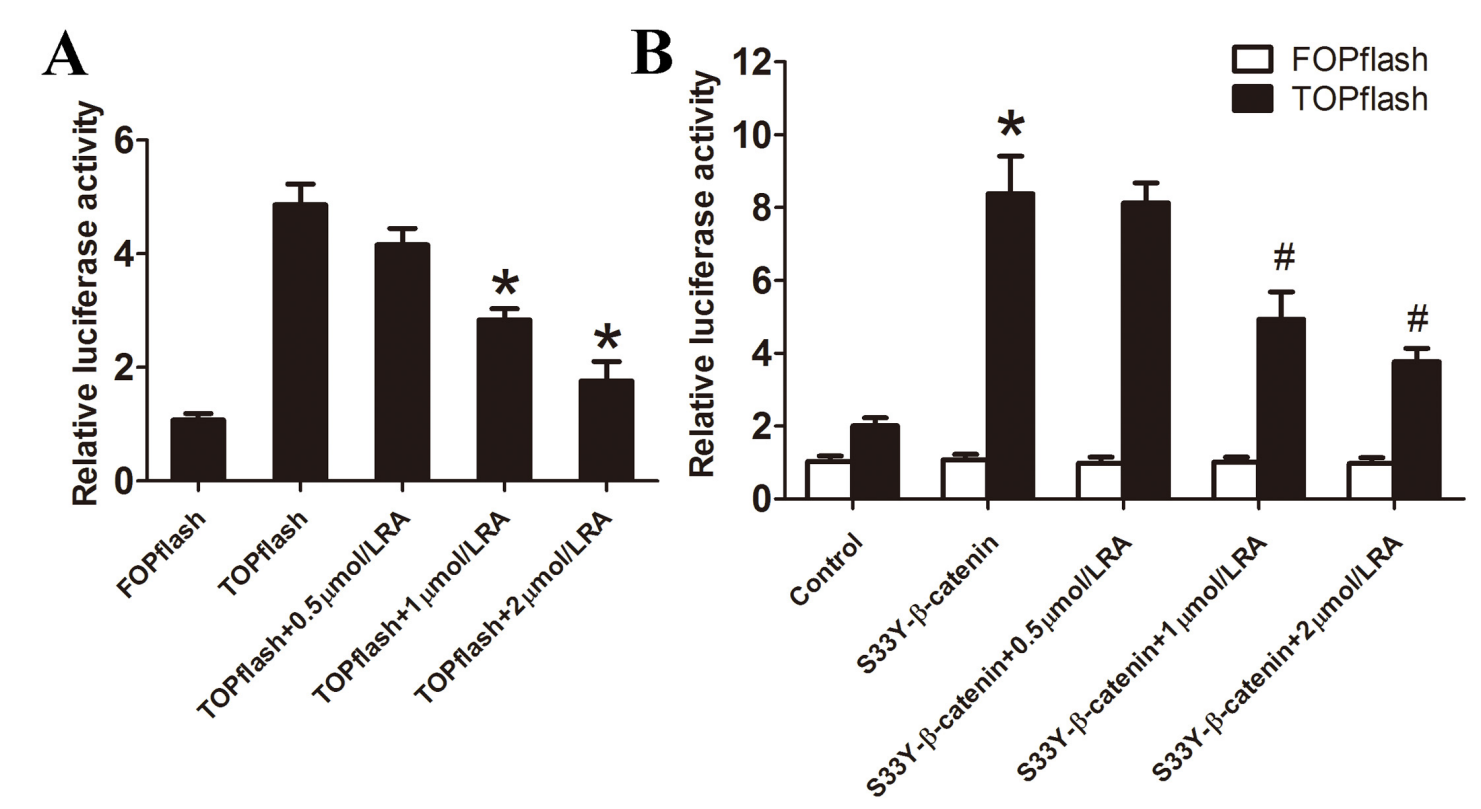
RA inhibits TCF/LEF-dependent transcriptional activity in PSCs (A) PSCs were transfected with TOPflash/FOPflash plasmids plus a pRL-SV40 as internal control and treated with different doses of RA (0.5, 1, 2 μmol/L). After 72h, using the dual-luciferase reporter assay system and normalized to Renilla luciferase activity to evaluate the luciferase activities. *p < 0.05 compared with the control group. (B) The S33Y-β-catenin plasmid and the reporter plasmid were co-transfected into PSCs, After 12h, cells were cultured with different doses of RA and measured the luciferase activities. *p < 0.05 compared with the empty vector group. ^#^ *p* < 0.05 compared with the S33Y-β-catenin group.

Day 5 PSCs were collected for immunofluorescence and Western blot analyses to establish the effects of RA on nuclear translocation of β-catenin. Treatment with RA significantly inhibited the nuclear translocation of β-catenin as outlined using immunofluorescence analysis (Fig 8A), further confirming inhibition of the Wnt/β-catenin signaling pathway by RA. Consistent with these results, Western blot experiments revealed marked down-regulation of Wnt 2 and nuclear β-catenin protein levels, while Axin 2 protein level was up-regulated in groups treated with RA, compared with the control group (Fig 8B). In addition, we evaluated the effect of RA on TCF/LEF-dependent transcriptional activity in PSCs by performing a luciferase reporter assay with TOPflash/FOPflash. Administration with RA markedly inhibited TCF/LEF-dependent transcriptional activity in PSCs (Fig 9A). Given β-catenin is a key mediator of Wnt/β-catenin pathway, we co-transfected the PSCs with S33Y-β-catenin plasmid plus the reporter plasmid. S33Y-β-catenin significantly increased the TCF/LEF-dependent transcriptional activity, compared with controls, and this increased activity was greatly suppressed in PSCs treated with RA. (Fig 9B).

## Discussion

The molecular mechanism of PSC activation, an important step in the development of pancreatic fibrosis, is largely unknown. One of the main characteristics of activation is a progressive decrease in the size and number of retinoid-containing fat droplets, in parallel with transition of PSCs towards a myofibroblastic phenotype [7]. RA exerts immunomodulatory effects and is used to treat acute promyelocytic leukemia and inflammatory disorders, such as psoriasis, acne, and rheumatoid arthritis [50]. Research on the effects of RA in cultured HSCs, a cell type playing a vital role in the development of liver fibrosis, has shown that retinoids are not only stored in these cells but also have various biological functions. Activated HSCs display a low proliferation rate following RA treatment [51] and a marked reduction in collagen I synthesis [52]. Hisamori and his team found RA can ameliorate liver fibrosis in mice through modulating cytokine production [35]. Further recent studies have indicated that RA maintain PSCs in a quiescent state and inhibit their activation [10,39]. However, the specific role of RA in PSCs and pancreatic fibrosis, especially chronic pancreatitis, is yet to be established. In the present study, we investigated the effect of RA using a mouse model of cerulein-induced CP *in vivo* and a cellular model of mouse PSCs *in vitro*. Our findings showed that RA not only inhibits activation of PSCs but also ameliorates progression of pancreatic fibrosis in experimental CP.

The mouse CP model involves repetitive administration of cerulein, an analog of the exocrine secretagogue cholecystokinin 8, leading to aggravated acinar injury, ECM deposition and fibrosis lesions. Along with progression of fibrosis, several mechanisms, including recruitment of immune cells and robust production of fibrogenic factors for tissue repair, are intensively elicited to prevent the pancreas from autodigestion of parenchyma [53]. In our study, H&E, Masson, α-SMA and Ki-67 staining experiments indicated reduced pancreatic damage and fibrosis following administration of RA *in vivo*, suggesting attenuation of the progression of pancreatic fibrosis induced by cerulein in CP mice. In addition, quantitative RT-PCR analysis of Amylase and CK19 outlined that RA has a negative effect in ADM, implicating reduction of acinar cell differentiation both *in vivo* and *in vitro*.

Myofibroblasts and cytokines, including TGF-β and PDGF, play a vital role in pancreatitis-associated fibrogenesis [54]. PSC activation is related to pancreatic fibrosis, and these activated cells are the main cellular source of collagen in CP [55]. Apte and colleagues found that PSCs produce collagen and other ECM proteins and respond to PDGF and TGF-β via increased proliferation and collagen synthesis [13]. Our results showed RA induces a significant reduction in the PDGFRβ, TGFβRII and collagen 1α1 mRNA levels, both *in vivo* and *in vitro*. Since PSCs express α-SMA upon pancreatic inflammation or injury, we further examined the expression patterns of α-SMA. Our Western blot and quantitative RT-PCR experiments revealed that RA down-regulates α-SMA expression in cerulein-induced CP mice but has no effect on α-SMA in primary cultured PSCs *in vitro*, and the different results consistent with earlier research [10,39]. Earlier, Huang and co-workers reported an RA-dependent decrease in expression of α-SMA in activated HSCs [56]. In view of the conflicting results on the expression patterns of α-SMA *in vivo* and *in vitro*, further studies are necessary to investigate whether these discrepancies are due to specific experiment design, inherent PSC performance or other factors. Nevertheless, our findings also revealed that RA suppresses progressive fibrosis of pancreas through modulating pro-inflammatory and pro-fibrotic mediators, and supporting the utility of RA as a promising therapeutic agent for pancreatic fibrosis.

The Wnt/β-catenin signaling pathway is well-established to be involved in regulating cell differentiation and proliferation [57]. Kim and his team found that Wnt/β-catenin signaling pathway associated with the RA-induced suppression of adipogenesis and cooperatively inhibit adipocyte differentiation [58]. RA can also inhibit the growth of cancer stem cells by suppression of this signaling pathway [59]. The team of Huang have suggested that the oncogenic activity of RA receptor gamma is exhibited via activation of the Wnt/β-catenin signaling pathway in cholangiocarcinoma [60]. We previously showed that the Wnt/β-catenin signaling pathway was activated during stimulation of PSCs *in vitro*. Wnt 2 was the only Wnt protein displaying an obvious increase after PSC activation. Protein levels of Wnt2 and β-catenin were shown to be significantly up-regulated in cerulein-induced mouse CP *in vivo*, and activation of PSCs *in vitro* suppressed upon silencing of β-catenin [31]. On the basis of these earlier findings, we investigated whether Wnt/β-catenin signaling pathway was involved in RA-mediated inhibition of pancreatic fibrosis and PSC activation. Upon administration of RA, expression levels of Wnt 2 and β-catenin and nuclear translocation of β-catenin were obviously down-regulated *in vivo* and *in vitro*, in accordance with previous data. Additionally, the expression of Axin 2 was significantly up-regulated *in vivo* and *in vitro* after RA adiministration. Notably, Wnt/β-catenin signaling pathway was up-regulated in experimental CP mice *in vivo* and during PSC activation *in vitro*, which was inhibited by RA. Additionally, RA suppressed PSC activation and collagen synthesis, and treatment with RA markedly promoted apoptosis, while inhibited proliferation and TCF/LEF-dependent transcriptional activity in PSCs. RA has already been widely used for differentiating therapy of acute promyelocytic leukaemia and oral administration of this drug results in good compliance in clinical. However, there exists many controversies about the role of RA in treatment of fibrotic diseases[61]. An excess of retinol is known to be hepatotoxic [62]. The team of Okuno showed that RA enhanced ECM production and exacerbated liver fibrosis by inducing the activation of TGF-β1, and the used dose in their study was greatly higher compared with the doses used in other studies [63–64], further demonstrating the unsuccessful application of RA in the treatment of liver fibrosis due to the hepatotoxicity caused by higher dose of RA. Our data and other previous findings indicated that RA could play a potential protective role in treatment of pancreatic fibrosis, but further studies are needed to explore the optimal dose for future clinical attempts.

The detailed mechanism of pancreatic fibrosis remains unclear at present. Data from our present study demonstrated that RA effectively exerts anti-fibrosis effects on CP induced by cerulein in mice. The profibrotic phenotype and proliferation of PSCs are inhibited and apoptosis of PSCs are promoted via suppression of the Wnt/β-catenin signaling pathway. Reduction of pro-inflammatory and pro-fibrotic mediator levels, including TGFβRII, PDGFRβ and collagen 1α1, positively prevent the development of pancreatic fibrosis and activation of PSCs. In summary, RA could function as a suppressor of pancreatic fibrosis in mice.
